# High-Level Exposure of Testosterone During Mouse Pregnancy Impairs the Offspring Social Behavior by Interrupting Neurexin–Neuroligin Binding

**DOI:** 10.3390/neurolint17080129

**Published:** 2025-08-16

**Authors:** Nan Yagishita-Kyo, Sosuke Yagishita

**Affiliations:** 1 Department of Pharmacology, Faculty of Medicine, Saitama Medical University, 38 Moro-Hongo, Moroyama-Machi, Iruma-Gun, Saitama 350-0495, Japan; 2 National Institute of Technology, Kisarazu College, 11-1, Kiyomidaihigashi 2-Chome, Kisarazu, Chiba 292-0041, Japan

**Keywords:** autism spectrum disorder, testosterone, neurexin, neuroligin

## Abstract

**Background/Objectives**: The onset of autism spectrum disorder (ASD) is thought to be related to fetal testosterone (TSTN) levels or the binding of neurexin (Nrxn) to neuroligin (Nlgn), as per some studies. However, the underlying molecular mechanisms remain unclear. We found that high concentrations of TSTN interrupt Nrxn–Nlgn binding in the neonatal brain, causing impaired social behavior. **Methods**: We reproduced high concentrations of TSTN in the womb by injecting TSTN into pregnant mice, followed by the quantification of Nrxn–Nlgn binding in the neonatal brain. We also explored the sociability and social novelty preferences of male and female offspring. **Results**: Nrxn–Nlgn binding in the neonatal brain decreased after TSTN injection. Furthermore, male mice showed impairment in social novelty, whereas female mice showed impairments in both social novelty and sociability following TSTN injection. **Conclusions**: This study revealed that high concentrations of TSTN during brain development interrupted Nrxn–Nlgn binding and led to impairments in social behavior.

## 1. Introduction

Testosterone (TSTN) is a key male hormone that plays an important role in fetal brain development [1]. In human studies, fetal TSTN levels inversely correlate with the frequency of eye contact and quality of social relationships in developing children [2,3]. In contrast, fetal TSTN levels are positively associated with narrow interests in children [3]. In animal studies, injection of an aromatase inhibitor into pregnant rats resulted in 1.25 times higher levels of serum TSTN and social interaction impairment in their female offspring [4]. Therefore, high concentrations of fetal TSTN are associated with later autism spectrum disorder (ASD) diagnosis [5,6]. The molecular mechanism of TSTN in social behavioral impairment remains largely unexplored; however, we found that TSTN affects neurexin (Nrxn) and neuroligin (Nlgn) interactions in vitro [7].

Nrxn and Nlgn, also known as “synaptic organizers,” are single transmembrane proteins localized at the pre- and post-synapses, respectively. Nrxn and Nlgn play essential roles in synapse differentiation and maturation [8,9,10], and these functions require trans-synaptic interactions between Nrxn and Nlgn [11,12]. Moreover, *Nrxn* and *Nlgn* gained attention as ASD genes as familial ASD mutations and copy number variations have been reported in both *Nrxn* and *Nlgn* [13,14,15,16,17]. In addition, Nlgn-knockout mice exhibit deficits in social interactions [18,19]. These studies suggest that normal expression of Nrxn and Nlgn at synaptic clefts is necessary for social function. Furthermore, the Nrxn–Nlgn trans-synaptic interaction is considered a crucial function in the regulation of social behavior [20].

Previously, we reported that TSTN directly binds to Nrxn and interrupts its binding to Nlgn in vitro [7]. Therefore, we hypothesized that high TSTN concentrations during brain development interrupt the Nrxn–Nlgn trans-synaptic interaction, leading to abnormal synaptic formation and impaired social behavior. This could explain the molecular mechanisms of TSTN in social behavioral deficits.

## 2. Materials and Methods

### 2.1. Animals

All animal experiments were performed in accordance with the regulations and guidelines for the care and use of experimental animals at Saitama Medical University and were approved by the institutional review committees. Pregnant C57BL/6J mice were purchased from the Jackson Laboratory Japan (Yokohama, Japan). They were 9 weeks old, and it was their first parturition. They were housed and maintained on a 12 h light/dark cycle and allowed ad libitum access to food and water. Following Hatanaka et al. [21], pregnant mice (E13–E15 days) were injected with 12.5 mg/kg TSTN solution or corn oil. Brain tissues from neonates born to the injected mice were subjected to immunoprecipitation (IP) assays. Conversely, neonates born to the injected mice were raised to 12 weeks of age and subjected to all behavioral experiments.

### 2.2. Antibodies and Chemicals

Anti-Nrxn3 antibody (HPA002727) was purchased from Atlas Antibodies (Bromma, Sweden). Anti-Nlgn (1/2/3/4) antibody (#129213) was purchased from Synaptic Systems (Goettingen, Germany). TSTN solution was purchased from Sigma-Aldrich (Tokyo, Japan).

### 2.3. Brain Sample Preparation and Co-IP with Nlgn

Neonatal C57BL/6J mice were anesthetized using sevoflurane and euthanized. Due to their early postnatal age, neonates were not sexed; therefore, the molecular analyses reflected pooled data from both sexes. The isolated brain tissues were divided into hippocampi and cortices, and frozen at –80 °C until use. The hippocampi were homogenized in 500 μL RIPA buffer (50 mM Tris-HCl buffer (pH 7.6), 150 mM NaCl, 1% NP-40, 0.5% sodium deoxycholate, and 0.1% sodium dodecyl sulfate) containing 1% phosphatase inhibitor cocktail (Nacalai Tesque Inc., Kyoto, Japan) and complete EDTA-free protease inhibitor cocktail (Sigma-Aldrich, Tokyo, Japan). After holding on ice for 10 min, hippocampus homogenates were centrifuged at 15,000× *g* for 20 min at 4 °C. The supernatants were immunoprecipitated at 4 °C overnight with anti-Nlgn 1/2/3/4 antibody, which were incubated for 1 h with Dynabeads Protein G (Thermo Fisher Scientific, Waltham, MA, USA). The magnetic beads were precipitated using a magnetic stand, followed by three washes with RIPA buffer. Protein samples were denatured with 15 μL Laemmli sample buffer (Bio-Rad Laboratories, Hercules, CA, USA) and boiled for 3 min.

### 2.4. Western Blotting

Co-IP samples from Section 2.3 were provided for Western blotting. They were loaded and separated on 5–10% precast gels (Nacalai Tesque Inc., Kyoto, Japan) by sodium dodecyl sulfate polyacrylamide gel electrophoresis and transferred onto nitrocellulose membranes (Bio-Rad Laboratories, Hercules, CA, USA). The membranes were blocked with 5% fat-free milk (MEGMILK SNOW BRAND Co., Ltd., Sapporo, Japan) for 1 h at room temperature and incubated overnight at 4 °C with the primary antibodies. The membranes were washed and probed with a horseradish peroxidase-conjugated goat anti-mouse/rabbit secondary antibody. Signals were detected using Chemi-Lumi One Super (Nacalai Tesque Inc., Kyoto, Japan) or ImmunoStar LD (Fujifilm Wako Pure Chemical Corporation, Osaka, Japan) with a ChemiDoc MP (Bio-Rad Laboratories, Hercules, CA, USA). The bands were detected and calculated using software (Image Lab (Version 6.1) and ImageJ (Version 1.54)). Relative signal intensities indicate Nrxn–Nlgn binding intensities. Consequently, they were calculated as the ratio of the Nrxn band intensity to the Nlgn band intensity (the amount of binding partner divided by the amount of precipitated protein) [7].

### 2.5. Behavioral Test

All behavioral tests were conducted during the daytime (13:00–16:00) when the lights were turned on. The brightness of the experimental room was identical to that of the breeding room, and the mice were moved to the experimental room at least 1 h before the tests. The chamber was washed with hot water and 70% ethanol and extracted every time after each session. The estrous cycle stage of female subjects is not monitored.

All behavioral tests were recorded using a web camera (Kyokuto-Electronics Inc., Osaka, Japan), and the videos were randomized by renaming the file names. The observers were blinded, and behavior was measured using a stopwatch.

#### 2.5.1. Three-Chamber Social Test

The three-chamber test was performed as described previously [22]. The three-chamber arena was a box (61 cm × 40 cm × 19 cm) made of a white acrylic board and divided into three equal compartments, where the mice were allowed to freely move between the compartments through doors on dividing walls.

The subject mice were habituated to the three-chamber arena for 5 min without any objects or animals. After habituation, mice were returned to their breeding cages. A stimulus mouse was placed under a wire cage (animal side) on one side of the chamber and a similar wire cage without a mouse was placed on the other side (object side). The subject mouse was placed in the middle chamber at the beginning of the test and was allowed to freely explore the three-chamber arena for 5 min. The sniffing time for each wire cage was measured to quantify sociability.

After the sociability session, the mouse was returned to its breeding cage. A new mouse was placed in the empty wire cage on the object side (novel animal side), while the already known mouse remained on the animal side (familiar animal side). The subject mouse was placed in the middle chamber and allowed to explore freely for 5 min. To quantify the social novelty, the sniffing time for each wire cage was measured.

To exclude the influence of sex on behavioral results, male and female subject mice were tested on the other day, and the sexes of the stimulus mice were equalized with those of the subject mice. The female subject mice were bred in the same cage and tested on the same day.

#### 2.5.2. Novel Object Recognition Test

A novel object recognition test was performed as described previously [23]. The subject mice were habituated to the three-chamber arena for 5 min without any objects. After habituation, mice were returned to their breeding cages. The same objects were placed on both sides (Object A), and the subject mice were placed in the middle chamber and allowed to explore freely for 5 min. After the same-object session, the subject mice were returned to their breeding cages. One side of Object A was replaced with a novel object (Object B). The subject mouse was placed in the middle chamber and allowed to explore freely for 5 min. To quantify novel object recognition, the sniffing times for Object A (familiar) and Object B (novel) were measured. For Object A and Object B, we used a small metal bucket or a glass bottle (both 9 cm in diameter). An object was designated as the novel one at random. Preliminary experiments confirmed no significant preference for either object.

#### 2.5.3. Y-Maze Test

The Y-maze test was performed as described previously [24]. The Y-maze apparatus (Hazai-ya, Tokyo, Japan) was a three-arm (A, B, or C) maze with equal angles among all arms (8 cm width), a bottom of 40 cm (length), and 15 cm height. The mice were tested individually by placing them in one arm of the maze and allowing them to move freely throughout the three arms for 10 min. The sequences and entries into each arm were recorded.

An accuracy rate was calculated as (the number of ‘successful’ alternations divided by the number of the total arm entries minus 2) × 100. A ‘successful’ alternation was defined as consecutive arm entries into the three different arms, such as, ABC, ACB, BCA, BAC, CAB, and CBA.

### 2.6. Statistical Analysis

**Figure 1:**
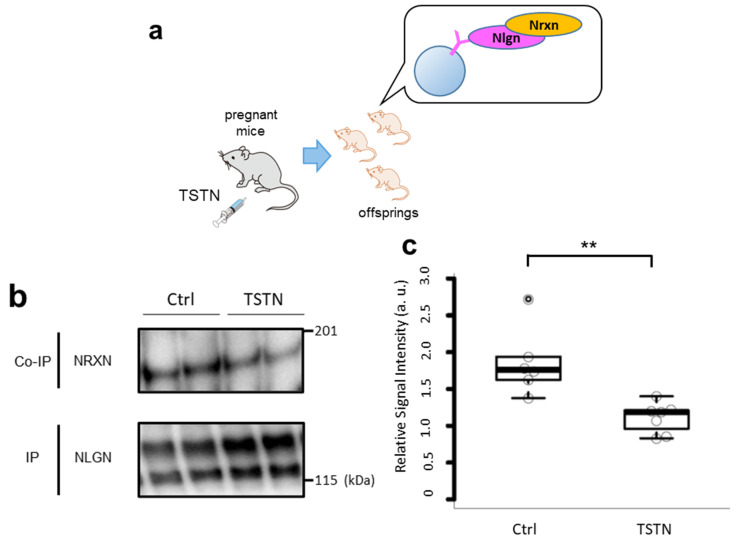
TSTN injection reduces Nrxn–Nlgn binding in the offspring’s brain. A schema of TSTN injection to the pregnant mice and co-IP of their offspring’s brain (**a**). Representative blots of co-IP assay with Nrxn and Nlgn. Both the upper and lower bands of Nlgn were quantitated (**b**). Box plot of the relative signal intensities of Nrxn/Nlgn (*n* = 7 and *n* = 7 from 3 pregnant females each, *p* = 0.008) (**c**). ** *p* < 0.01. These data reflect pooled sexes.

**Figure 2:**
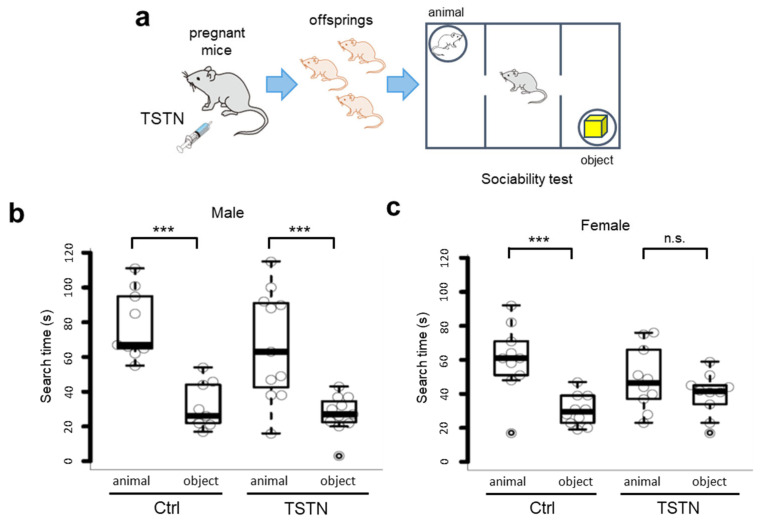
Search time of sociability test. A schema of TSTN injection to the pregnant mice and sociability test (**a**). Box plot of search time of male offspring (*n* = 9 and *n* = 11 from 3 pregnant females each, *p* = 0.0001749, *p* = 0.0004428, by Tukey’s HSD) (**b**). Box plot of search time of female offspring (*n* = 10 and *n* = 10 from 3 pregnant females each, *p* = 0.0007076, *p* = 0.5642661, by Tukey’s HSD) (**c**). *** *p* < 0.001, n.s., not significant.

**Figure 3:**
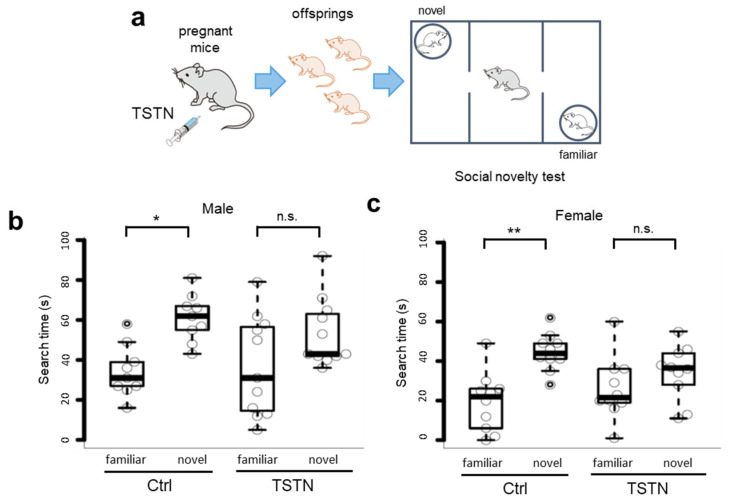
Search time of social novelty test. A schema of TSTN injection to the pregnant mice and social novelty test (**a**). Box plot of search time of male offspring (*n* = 9 and *n* = 11 from 3 pregnant females each, *p* = 0.0149682, and *p* = 0.1504518, by Tukey’s HSD) (**b**). Box plot of search time of female offspring (*n* = 10 and *n* = 10 from 3 pregnant females each, *p* = 0.0011853, and *p* = 0.5449553, by Tukey’s HSD) (**c**). * *p* < 0.05, ** *p* < 0.01, and n.s., not significant.

**Figure 4:**
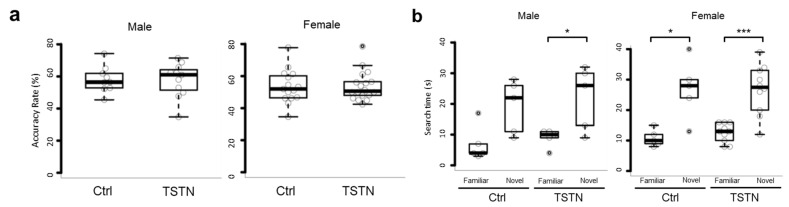
Box plot of accuracy rate of Y-maze test (*n* = 9, *n* = 11, *n* = 15, and *n* = 18 from 3 pregnant females each, *p* = 0.91529 and *p* = 0.96327) (**a**). Box plot of search time of novel object recognition test (*n* = 5, *n* = 5, *n* = 5, and *n* = 10 from 3 pregnant females each, *p* = 0.05752 *p* = 0.03289, *p* = 0.0289, and *p* = 0.0003, by Tukey’s HSD) (**b**). * *p* < 0.05, *** *p* < 0.001.

All statistical calculations were performed using the R software (Version 4.5.1) (R core Team, Vienna, Austria. All data were presented by mean ± SEM. Student’s *t*-test (Figure 1) was performed. Multiple analyses were performed using two-way repeated ANOVA followed by Tukey’s HSD (Figures 2–4). A paired *t*-test (Figure 4) was performed. A two-way mixed-design ANOVA was performed to examine the effects of treatment and stimulus type. In this design, stimulus (object vs. animal or familiar vs. novel) was treated as a within-subject factor, and the treatment group (control vs. TSTN or male vs. female) as a between-subject factor. All results from the two-way mixed-design ANOVA analyses are summarized in the Supplementary Tables S1–S3.

## 3. Results

### 3.1. Nrxn and Nlgn Binding Is Interrupted by TSTN Injection

We injected TSTN or vehicle into pregnant mice to intensify TSTN elevation in the womb (Figure 1a). We analyzed the binding intensities of Nrxn and Nlgn in the TSTN-injected neonatal brains using a co-IP assay (Figure 1b). According to co-IP results, Nrxn–Nlgn binding was reduced by approximately 75% following maternal TSTN injection (Figure 1c).

### 3.2. Sociability Searching Time Does Not Differ in Female Offspring from TSTN-Injected Mother

We raised the neonates to 12 weeks old and subjected them to sociability tests (Figure 2a). According to the two-way ANOVA results, there was no significant main effect of treatment (offspring born to the vehicle-injected mother (Ctrl) vs. offspring born to the TSTN mother (TSTN)), F(1, 18) = 1.836, and *p* = 0.192 in male offspring, and there was also no significant main effect of treatment (Ctrl vs. TSTN), F(1, 18) = 0.052, and *p* = 0.822 in female offspring. However, when comparing males and females in the TSTN group, two-way ANOVA revealed a significant interaction between stimulus type (object vs. animal) and sex (male vs. female), F(1, 19) = 5.222, *p* = 0.03396 (Supplementary Table S1). Both male and female offspring born to Ctrl mothers and male offspring born to TSTN-injected mothers spent significantly more time searching for the animal side than for the object side (Figure 2b). Notably, there were no differences in the animal/object search time between TSTN female offspring (Figure 2c).

### 3.3. Social Novelty Searching Time Does Not Differ in Offspring from TSTN-Injected Mother

Next, we performed the social novelty tests (Figure 3a). Interestingly, when comparing male and female offspring in the TSTN group, the two-way ANOVA revealed a significant main effect of sex (F(1, 19) = 5.469, *p* = 0.0304 (Supplementary Table S2). The control offspring exhibited significant differences between novel and familiar animal sides. They searched for a novel animal longer than for a familiar animal. In contrast, both male and female TSTN offspring exhibited little difference between the novel and familiar animal sides (Figure 3b,c).

### 3.4. TSTN Does Not Affect Working Memory in Offspring

To confirm whether the behavioral alterations in the TSTN offspring were confined to sociality, we performed Y-maze and novel object recognition tests. According to the Y-maze test results, the accuracy rate was consistent across the control and TSTN groups in both male and female offspring (Figure 4a). In addition, in the novel object recognition test, the tendency to search for novel objects for a longer period than for familiar objects was consistent (Figure 4b). Two-way ANOVA revealed a significant main effect of stimulus type (familiar vs. novel) *F*(1, 8) = 16.782, and *p* = 0.00345, in male offspring. At this time, there was no significant main effect of treatment (Ctrl vs. TSTN), *F*(1, 8) = 0.441, and *p* = 0.525. Similarly, in female offspring, there was no significant main effect of treatment (Ctrl vs. TSTN) and *F*(1, 13) = 0.084, *p* = 0.777, and the significant main effect of stimulus type (familiar vs. novel) was *F*(1, 13) = 41.481 and *p* < 0.00001 (Supplementary Table S3).

## 4. Discussion

According to previous studies, Nrxn have six LNS domains. The LNS domain is named after laminin, neurexin, and sex hormone-binding globulin (SHBG), based on its structural similarity [25]. Therefore, Nrxn binds directly to TSTNs such as SHBG. Furthermore, this binding interrupts Nrxn–Nlgn interaction in vitro [7]. Based on these findings, it is considered that in the present study as well, TSTN may interfere with Nrxn–Nlgn binding by directly binding to Nrxn.

In mice, fetal TSTN levels increase from E13 to a peak at E17 and are higher in male fetuses than in female fetuses [26,27]. This TSTN surge is a normal physiological process during male fetal development, and TSTN of fetal origin contributes to increased concentrations in the amniotic fluid. To intensify the TSTN surge, we injected TSTN at E13–E15. Subcutaneous administration of 0.3 mg TSTN to pregnant mice led to abnormal synaptic instability and aberrant morphology of dendritic spines in the brains of their offspring [21]. These synaptic abnormalities are thought to be related to abnormalities in social behaviors. In the present study, we used the corresponding TSTN administration methods. As fetal and amniotic TSTN levels were not directly measured in this study, the actual exposure of the developing fetus remains uncertain. Furthermore, we did not directly monitor the estrous cycle stage of female offspring. This is a limitation that needs to be addressed in future studies.

Brain masculinization in rodents is distinct from that observed in humans. Sex differences in the brains of male mice are influenced by estrogens derived from the neural aromatization of TSTN [28,29,30]. However, social behavior impairment was discovered following aromatase inhibitor injection in pregnant rats, which led to an increase in serum TSTN [4]. Administration of TSTN to pregnant rats also impairs the social behavior of their offspring [31]. Therefore, social and behavioral deficits seem to be directly caused by the abundance of fetal TSTN. Thus, the effect of TSTN on social ability may be due to interference with Nrxn–Nlgn binding. Although our study did not directly address the aromatization of TSTN, future studies should consider the potential contribution of aromatization to the behavioral outcomes observed here.

According to previous studies, the children of mothers with high levels of fetal TSTN are affected by social interactions [2,3]. In the early development period, the plasma TSTN concentration was approximately 2 nM in neonatal male mice, whereas it was less than 0.5 nM in neonatal female mice [32,33,34]. In this study, we aimed to increase the concentration of fetal TSTN to investigate its impact on social behavior. Under these conditions, female offspring were more strongly influenced by TSTN injection because the gap between native TSTN concentration and exogenous TSTN administration was wider in female offspring than in male offspring. Thus, the impairment in social ability caused by TSTN injection may be stronger in female offspring. Although ASD is more frequently diagnosed in males, previous studies reported that females with ASD often present with more severe symptoms or greater impairments, possibly due to diagnostic biases or underlying biological differences [27,35]. Therefore, females might be more sensitive to certain prenatal exposures. However, our explanation remains speculative and should be interpreted with caution. Further investigations are needed to clarify the mechanisms underlying sex-specific vulnerability.

We acknowledge that the absence of circulating TSTN and estradiol measurements in the offspring limits our ability to determine whether the hypothalamic–pituitary–gonadal axis is altered. Similarly, without anxiety-related assessments (e.g., open field or elevated plus maze), we cannot exclude the influence of altered emotional reactivity on social behavior. Future studies incorporating both endocrine profiling and formal anxiety tests are essential to elucidate the mechanisms underlying the behavioral phenotypes reported here.

## Data Availability

Raw data were generated at Saitama Medical University. The derived data supporting the findings of this study are available from the corresponding author, N.Y.-K. on request.

## Supplementary Materials

The following supporting information can be downloaded at: https://www.mdpi.com/article/10.3390/neurolint17080129/s1, Supplementary Table S1. Summary of two-way ANOVA for three-chamber sociality test; Supplementary Table S2. Summary of two-way ANOVA for three-chamber social novelty test; Supplementary Table S3. Summary of two-way ANOVA for object novelty test.

## Abbreviations

The following abbreviations are used in this manuscript:

ASD	Autism spectrum disorder
TSTN	Testosterone
Nrxn	Neurexin
Nlgn	Neuroligin
IP	Immunoprecipitation

## References

[1] Collignon A., Dion-Albert L., Ménard C., Coelho-Santos V. (2024). Sex, hormones and cerebrovascular function: From development to disorder. Fluids Barriers CNS.

[2] Lutchmaya S., Baron-Cohen S., Raggatt P. (2002). Foetal testosterone and eye contact in 12 month old infants. Infant. Behav. Dev..

[3] Knickmeyer R., Baron-Cohen S., Raggatt P., Taylor K. (2005). Foetal testosterone, social cognition, and restricted interests in children. J. Child Psychol. Psych..

[4] Xu X.J., Zhang H.F., Shou X.J., Li J., Jing W.L., Zhou Y., Qian Y., Han S.P., Zhang R., Han J.S. (2015). Prenatal hyperandrogenic environment induced autistic-like behavior in rat offspring. Physiol. Behav..

[5] Baron-Cohen S., Lombardo M.V., Auyeung B., Ashwin E., Chakrabarti B., Knickmeyer R. (2011). Why are autism spectrum conditions more prevalent in males?. PLoS Biol..

[6] Baron-Cohen S., Auyeung B., Nørgaard-Pedersen B., Hougaard D.M., Abdallah M.W., Melgaard L., Cohen A.S., Chakrabarti B., Ruta L., Lombardo M.V. (2015). Elevated fetal steroidogenic activity in autism. Mol. Psychiatry.

[7] Yagishita-Kyo N., Ikari Y., Uekita T., Shinohara A., Koshimoto C., Yoshikawa K., Maruyama K., Yagishita S. (2021). Testosterone interrupts binding of Neurexin and Neuroligin that are expressed in a highly socialized rodent, Octodon degus. Biochem. Biophys. Res. Commun..

[8] Graf E.R., Zhang X., Jin S.X., Linhoff M.W., Craig A.M. (2004). Neurexins induce differentiation of GABA and glutamate postsynaptic specializations via neuroligins. Cell.

[9] Prange O., Wong T.P., Gerrow K., Wang Y.T., El-Husseini A. (2004). A balance between excitatory and inhibitory synapses is controlled by PSD-95 and neuroligin. Proc. Natl. Acad. Sci. USA.

[10] Kang Y., Zhang X., Dobie F., Wu H., Craig A.M. (2008). Induction of GABAergic postsynaptic differentiation by alpha-neurexins. J. Biol. Chem..

[11] Ko J., Zhang C., Araç D., Boucard A.A., Brunger A.T., Südhof T.C. (2009). Neuroligin-1 performs neurexin-dependent and neurexin-independent functions in synapse validation. EMBO J..

[12] Gokce O., Südhof T.C. (2013). Membrane-tethered monomeric neurexin LNS-domain triggers synapse formation. J. Neurosci..

[13] Jamain S., Quach H., Betancur C., Råstam M., Colineaux C., Gillberg I.C., Soderstrom H., Giros B., Leboyer M., Gillberg C. (2003). Paris Autism Research International Sibpair Study. Mutations of the X-linked genes encoding neuroligins NLGN3 and NLGN4 are associated with autism. Nat. Genet..

[14] Laumonnier F., Bonnet-Brilhault F., Gomot M., Blanc R., David A., Moizard M.P., Raynaud M., Ronce N., Lemonnier E., Calvas P. (2004). X-linked mental retardation and autism are associated with a mutation in the NLGN4 gene, a member of the neuroligin family. Am. J. Hum. Genet..

[15] Julie G., Tabrez J.S., Peng H., Daisaku Y., Fadi F.H., Nathalie C., Mathieu L., Dan S., Anne N., Ronald G.L. (2011). Truncating mutations in NRXN2 and NRXN1 in autism spectrum disorders and schizophrenia. Hum. Genet..

[16] Sanders S.J., Ercan-Sencicek A.G., Hus V., Luo R., Murtha M.T., Moreno-De-Luca D., Chu S.H., Moreau M.P., Gupta A.R., Thomson S.A. (2011). Multiple recurrent de novo CNVs, including duplications of the 7q11.23 Williams syndrome region, are strongly associated with autism. Neuron.

[17] Pohl T.T., Hörnberg H. (2022). Neuroligins in neurodevelopmental conditions: How mouse models of de novo mutations can help us link synaptic function to social behavior. Neuronal Signal..

[18] Radyushkin K., Hammerschmidt K., Boretius S., Varoqueaux F., El-Kordi A., Ronnenberg A., Winter D., Frahm J., Fischer J., Brose N. (2009). Neuroligin-3-deficient mice: Model of a monogenic heritable form of autism with an olfactory deficit. Genes Brain Behav..

[19] Jamain S., Radyushkin K., Hammerschmidt K., Granon S., Boretius S., Varoqueaux F., Ramanantsoa N., Gallego J., Ronnenberg A., Winter D. (2008). Reduced social interaction and ultrasonic communication in a mouse model of monogenic heritable autism. Proc. Natl. Acad. Sci. USA.

[20] Connor S.A., Ammendrup-Johnsen I., Chan A.W., Kishimoto Y., Murayama C., Kurihara N., Tada A., Ge Y., Lu H., Yan R. (2016). Altered Cortical Dynamics and Cognitive Function upon Haploinsufficiency of the Autism-Linked Excitatory Synaptic Suppressor MDGA2. Neuron.

[21] Hatanaka Y., Wada K., Kabuta T. (2015). Abnormal instability, excess density, and aberrant morphology of dendritic spines in prenatally testosterone-exposed mice. Neurochem. Int..

[22] Kaidanovich-Beilin O., Lipina T., Vukobradovic I., Roder J., Woodgett J.R. (2011). Assessment of social interaction behaviors. J. Vis. Exp..

[23] Nomoto M., Ohkawa N., Nishizono H., Yokose J., Suzuki A., Matsuo M., Tsujimura S., Takahashi Y., Nagase M., Watabe A.M. (2016). Cellular tagging as a neural network mechanism for behavioural tagging. Nat. Commun..

[24] Yagishita S., Suzuki S., Yoshikawa K., Iida K., Hirata A., Suzuki M., Takashima A., Maruyama K., Hirasawa A., Awaji T. (2017). Treatment of intermittent hypoxia increases phosphorylated tau in the hippocampus via biological processes common to aging. Mol. Brain.

[25] Rudenko G., Hohenester E., Muller Y.A. (2001). LG/LNS domains: Multiple functionsone business end?. Trends Biochem. Sci..

[26] Pointis G., Latreille M.T., Mignot T.M., Janssens Y., Cedard L. (1979). Regulation of testosterone synthesis in the fetal mouse testis. J. Steroid Biochem..

[27] Romano E., Cosentino L., Laviola G., De Filippis B. (2016). Genes and sex hormones interaction in neurodevelopmental disorders. Neurosci. Biobehav. Rev..

[28] Baum M.J. (1979). Differentiation of coital behavior in mammals: A comparative analysis. Neurosci. Biobehav. Rev..

[29] MacLusky N.J., Naftolin F. (1981). Sexual differentiation of the central nervous system. Science.

[30] Bakker J., Brand T., van Ophemert J., Slob A.K. (1993). Hormonal regulation of adult partner preference behavior in neonatally ATD-treated male rats. Behav. Neurosci..

[31] Erdogan M.A., Bozkurt M.F., Erbas O. (2023). Effects of prenatal testosterone exposure on the development of autism-like behaviours in offspring of Wistar rats. Int. J. Dev. Neurosci..

[32] Jean-Faucher C., Berger M., de Turckheim M., Veyssiere G., Jean C. (1978). Developmental patterns of plasma and testicular testosterone in mice from birth to adulthood. Acta Endocrinol..

[33] Elliot S.J., Berho M., Korach K., Doublier S., Lupia E., Striker G.E., Karl M. (2007). Gender-specific effects of endogenous testosterone: Female α-estrogen receptor-deficient C57Bl/6J mice develop glomerulosclerosis. Kidney Int..

[34] Cruz C.D., Kinnear H.M., Hashim P.H., Wandoff A., Nimmagadda L., Chang F.L., Padmanabhan V., Shikanov A., Moravek M.B. (2023). A mouse model mimicking gender-affirming treatment with pubertal suppression followed by testosterone in transmasculine youth. Hum. Reprod..

[35] Lai M.C., Baron-Cohen S., Buxbaum J.D. (2015). Understanding autism in the light of sex/gender. Mol. Autism..

