# Supplementary material for: High-Level Exposure of Testosterone During Mouse Pregnancy Impairs the Offspring Social Behavior by Interrupting Neurexin–Neuroligin Binding
Source: Neurol Int. 2025 Aug 16;17(8):129. doi: 10.3390/neurolint17080129 (PMC12388718; doi:10.3390/neurolint17080129)
Supplement: Supplementary file 1 [file neurolint-17-00129-s001.zip › neurolint-3740559-supplementary.pdf]

a Male offsprings

Source of Variation	SS (Sum of Squares)	df	MS (Mean Square)	F-value	p-value
Factor A (object vs. animal)	18404	1	18404	35.038	1.33e-05 ***
Factor B (Ctrl vs. TSTN)	637	1	636.8	1.836	0.192
Interaction (A × B)	130	1	130	0.248	0.625
Total	9455	18			

b Female offsprings

Source of Variation	SS (Sum of Squares)	df	MS (Mean Square)	F-value	p-value
Factor A (object vs. animal)	3881	1	3881	14.717	0.00121 **
Factor B (Ctrl vs. TSTN)	12	1	12.1	0.052	0.822
Interaction (A × B)	1102	1	1102	4.181	0.05579
Total	4747	18			

c Male vs. Female offsprings (TSTN injection)

Source of Variation	SS (Sum of Squares)	df	MS (Mean Square)	F-value	p-value
Factor A (object vs. animal)	6638	1	6638	14.287	0.00127 **
Factor B (Male vs. Female)	82	1	81.6	0.228	0.638
Interaction (A × B)	2426	1	2426	5.222	0.03396 *
Total	8827	19			

Supplementary Table 1. Summary of two-way ANOVA for three-chamber sociality test. * $p < 0.05$, ** $p < 0.01$, *** $p < 0.001$and n.s., not significant

a Male offsprings

Source of Variation	SS (Sum of Squares)	df	MS (Mean Square)	F-value	p-value
Factor A (familiar vs. novel)	4537	1	4537	16.574	0.000717 ***
Factor B (Ctrl vs. TSTN)	66	1	66.2	0.178	0.678
Interaction (A × B)	266	1	266	0.971	0.337
Total	4927	18			

b Female offsprings

Source of Variation	SS (Sum of Squares)	df	MS (Mean Square)	F-value	p-value
Factor A (familiar vs. novel)	2789	1	2788.9	12.446	0.0024 **
Factor B (Ctrl vs. TSTN)	36.1	1	36.1	0.239	0.631
Interaction (A × B)	722	1	722.5	3.224	0.0894
Total	4034	18			

c Male vs. Female offsprings (TSTN injection)

Source of Variation	SS (Sum of Squares)	df	MS (Mean Square)	F-value	p-value
Factor A (familiar vs. novel)	1672	1	1672	6.543	0.0192 *
Factor B (Male vs. Female)	2369	1	2368.6	5.469	0.0304 *
Interaction (A × B)	186	1	186.4	0.729	0.4037
Total	4855	19			

Supplementary Table 2. Summary of two-way ANOVA for three-chamber social novelty test. * $p < 0.05$, ** $p < 0.01$, *** $p < 0.001$and n.s., not significant

a Male offsprings

Source of Variation	SS (Sum of Squares)	df	MS (Mean Square)	F-value	p-value
Factor A (familiar vs. novel)	793.8	1	793.8	16.782	0.00345 **
Factor B (Ctrl vs. TSTN)	28.8	1	28.8	0.441	0.525
Interaction (A × B)	0.8	1	0.8	0.017	0.89974
Total	378.4	8			

b Female offsprings

Source of Variation	SS (Sum of Squares)	df	MS (Mean Square)	F-value	p-value
Factor A (familiar vs. novel)	1642.8	1	1642.8	41.481	2.2e-05 ***
Factor B (Ctrl vs. TSTN)	3.8	1	3.75	0.084	0.777
Interaction (A × B)	7.3	1	7.3	0.186	0.674
Total	514.8	13			

Supplementary Table 3. Summary of two-way ANOVA for object novelty test. * $p < 0.05$, ** $p < 0.01$, *** $p < 0.001$and n.s., not significant
